# Comparison of Pulegone and Estragole Chemotypes Provides New Insight Into Volatile Oil Biosynthesis of *Agastache rugosa*

**DOI:** 10.3389/fpls.2022.850130

**Published:** 2022-04-06

**Authors:** Jingjie Dang, Guyin Lin, Licheng Liu, Peina Zhou, Yongfang Shao, Shilin Dai, Mengru Sang, Zheng Jiang, Chanchan Liu, Qinan Wu

**Affiliations:** ^1^College of Pharmacy, Nanjing University of Chinese Medicine, Nanjing, China; ^2^Collaborative Innovation Center of Chinese Medicinal Resources Industrialization, Nanjing, China

**Keywords:** *Agastache rugosa*, transcriptome sequencing, chemotypes, monoterpenoid biosynthesis, phenylpropanoids biosynthesis

## Abstract

The aerial parts of *Agastache rugosa* are rich in essential oils containing monoterpenoids, phenylpropanoids, and aromatic compounds. These are used as herbs, perfume plants, and ornamental plants. Based on the difference in the constituents of the essential oil, *A. rugosa* is divided into pulegone and estragole chemotypes, but the mechanism of key metabolite biosynthesis in these two *A. rugosa* chemotypes remains unclear. In this study, we compared the morphological differences, metabolite constituents, and transcriptomic data between the two chemotypes of *A. rugosa*. Monoterpenoid was the main compound in the pulegone chemotype, and phenylpropanoid was the main compound in the estragole chemotype; however, limonene was detected in both chemotypes. Furthermore, 46 genes related to pulegone and estragole biosynthesis were identified. *Limonene synthase*, *limonene-3-hydroxylase*, and *isopiperitenol dehydrogenase* were upregulated in the pulegone chemotype, while *phenylalanine ammonia-lyase*, *4-coumarate: CoA ligase*, *CYP73A*, *coumaroyl-aldehyde dehydrogenase*, and *eugenol synthase* were downregulated in the pulegone chemotype. We identified chavicol methyl transferase and limonene-3-hydroxylase in *A. rugosa.* This work not only provides the difference in morphology and metabolites in pulegone and estragole chemotypes, but also offers a molecular mechanism of volatile oil biosynthesis, which could be a basis for specialized metabolites in specialized chemotypes.

## Introduction

*Agastache rugosa* (Fisch. & C.A.Mey.) Kuntze (*A. rugosa*), belonging to the family Lamiaceae is one of the best-known genera for its medicinal properties and economically important aromatic oils. *A. rugosa* has traditionally been used to treat naupathia, emesis, and to dispel dampness (Cao et al., 2017). Studies have indicated that *A. rugosa* has a variety of pharmacological actions, such as antifungal, anti-oxygenation, and anti-inflammatory activities. The aerial parts of *A. rugosa* and their constituent compounds have the potential for the development of natural nematicides (Li et al., 2013).

Based on the differences in chemical compounds within the same species, the plants were divided into different chemotypes. Chemotypes are common in plants, and most of them are concentrated in the Lamiaceae and Asteraceae families, such as *Artemisia annua* and *Mentha canadensis Linnaeus* (Šarić-Kundalić et al., 2009; Czechowski et al., 2018). The morphological differences between a variety of chemotypes were mainly in leaf type, trichome density, plant height, and internode length (Czechowski et al., 2018; Wêglarz et al., 2020). The existence of a chemotype has been widely reported, but the mechanism of its generation is not clear. Research on the chemotypes of *A. rugosa* has mainly focused on differences in chemical composition. The chemical composition of the essential oils obtained from the aerial parts (stems, flowers, and leaves) of *A. rugosa* grown in different regions has been the subject of some studies (Skakovskii et al., 2010; Mo and Ma, 2011). Li et al. found that pulegone, estragole, and methyl eugenol were the principal components of *A. rugosa* aerial parts collected from Zhejiang, Hubei, and Henan province of China (Li et al., 2013) which were represented by chemotypes. However, studies on morphological and biosynthesis differences in the chemotypes of *A. rugosa* have not been conducted.

To better understand the differences in the chemotypes of *A. rugosa* and the molecular mechanism of their formation, we systematically compared morphological data and volatile oil components in the pulegone (ArP) and estragole (ArE) chemotypes of *A. rugosa*, the genes related to pulegone and estragole biosynthesis were identified using *de novo* transcriptome sequencing, and the functions of L3OH and CVOMT were verified *in vitro*. Quantitative real-time PCR (qRT-PCR) further validated the gene expression levels of the two chemotypes in *A. rugosa*.

## Materials and Methods

### Plant Materials and Growth Condition

The seeds of *Agastache rugosa* (Fisch. et Mey.) O.Ktze. were collected from Jiangxi, China, and grown at Nanjing University of Chinese Medicine, Jiangsu, China. The growth condition was described as Liu L. et al. (2021).

### Trichome Density Measurements

Trichome density was quantified on the abaxial surface of the terminal leaflets of leaves 3–5 (counted from the apical meristem). Trichomes were visualized using a stereomicroscope (SetREO Discovery. V20, Zeiss, Germany). Images were recorded using Axio Vision 4.7 software (Carl Zeiss Ltd., Herts., United Kingdom). The trichome number was counted manually across a measured leaf area (automatically calculated by the software), and the average (mean) trichome density was then calculated for the whole leaf.

### Simultaneous Distillation Extraction of Essential Oils

Leaves, stems, and roots were harvested at the lush stage (60 day after germination). Five grams of fresh sample material was transferred to a 500 mL round bottom flask with 100 mL of deionized water for subsequent simultaneous distillation extraction using a modified Likens-Nickerson apparatus and n-hexane as the carrier solvent. An aliquot of the n-hexane fraction, which contained volatile oil constituents, was transferred to a 2 mL glass vial for further analysis.

### Essential Oils Analysis

Gas chromatography-mass spectrometry (GC-MS) was performed using a 6890N GC interfaced with 5973 inert MS instrument (Agilent Technologies, Santa Clara, CA, United States) and chromatographic column (Agilent 19091S-433-HP-5ms, 30 m × 250 μm × 0.25 μm). GC/MS procedure: kept at 50°C for 3 min, elevated to 90°C at 3°C/min, and then raised to 220°C at 10°C/min, and maintained for 5 min. The injection volume was 1.0 μL and splitless. The temperature of the injection port was 220°C, the EI ion source was 230°C, the electron energy was 70 eV, and the connection line temperature was 150°C. The MS scan range (m/z) was 50–500 m/z. The scanning speed was 2 times/s, and the delay time was 2.5 min. For analysis, essential oils dissolved in n-hexane were directly injected. Metabolites were identified based on chromatographic and spectral comparisons against the NIST/EPA/NIH mass spectral library version 2.0 and standards. The percentage of each chemical constituent in the essential oil was determined by the normalization of the peak.

Gas chromatography (GC) was performed using an 8860 GC (Agilent Technologies, Santa Clara, CA, United States) and chromatographic column (Agilent 19091 J-413-HP-5, 30 m × 0.32 mm × 0.25 μm). The GC procedure was maintained at 50°C for 3 min, elevated to 90°C at 3°C/min, and then raised to 220°C at 10°C/min, and maintained for 5 min. The injection volume was 1.0 μL and splitless. The temperature of the injection port was 250°C, and the flame ionization detector temperature was 250°C.

Chiral-GC was performed using an 8860 GC (Agilent Technologies, Santa Clara, CA, United States) and chromatographic column (Agilent 112-5532-DB-5ms, 30 m × 0.25 mm × 0.25 μm). Chiral-GC procedure: kept at 40°C for 0 min, elevated to 90°C at 2°C/min, and then raised to 120°C at 1°C/min, and maintained for 2 min. The injection volume was 2.0 μL and splitless. The temperature of the injection port was 250°C, and the flame ionization detector temperature was 250°C.

### Total RNA Extraction, mRNA Library Construction, and Sequencing

Leaf samples with estragole or pulegone as the first principal component of essential oil were collected separately and quickly frozen in liquid nitrogen. Three biological replicates of the leaves of each chemotype were collected for RNA sequencing (RNA-Seq), each consisting of four young and two old leaves from the same plant. Total RNA was extracted using TRIzol Reagent (Sigma, United States). RNA samples of *A. rugosa* were separately sequenced using the BGISEQ-500 Transcriptome platform, in which the mRNA was purified from total RNA using Oligo (dT)-attached magnetic beads, and the PCR products were purified using Ampure XP Beads and library quality was assessed on the Agilent Technologies 2100 bioanalyzer. The final library was amplified with phi29 to form a DNA nanoball (DNB), which had more than 300 copies of one molecule, DNBs were loaded into the patterned nanoarray, and pair-end 100 bases reads were generated on the BGIseq500 platform (BGI-Shenzhen, China).

### Assembly, Annotation, and Differential Gene Expression Analysis

The raw data were cleaned by removing low-quality and adapter reads. Transcriptome *de novo* assembly of clean reads was conducted using Trinity (Love et al., 2014). After assembly, unigenes were obtained and annotation was conducted using the following public databases: GO, KEGG, KOG, NR, NT, Pfam, PRG, and Swissprot. Differential gene expression analyses of the two chemotypes were performed using genes with an adjusted *P*-value ≤ 0.5 and were considered to be differentially expressed. According to the results of differential gene expression, the PHYPER function in R software was used for the enrichment analysis.

### Quantitative Real-Time PCR Analysis

The qRT-PCR analysis was conducted to verify the expression of selected genes in *A. rugosa.* The Hiscript III 1st Strand cDNA Synthesis Kit (+gDNA wiper) (Vazyme Biotech, Nanjing, China) was used with 500 ng of total RNA to synthesize first-strand cDNA. The qRT-PCR reactions were conducted using the ChamQ Universal SYBR qPCR Master Mix Kit (Vazyme Biotech, Nanjing, China). The reaction system and steps were performed according to the manufacturer’s instructions. The *A. rugosa* actin gene was used as a control to normalize the relative expression levels of the target genes. All results are representative of the three independent experiments. The primers used for qRT-PCR are listed in Supplementary Table 1.

### Isolation of Full-Length Limonene-3-Hydroxylase and Chavicol Methyl Transferase

Single-strand cDNA was used as the template for PCR amplification of the target cDNA with 2 × PrimeSTAR^®^ Max DNA Polymerase (Takara, Japan) and gene-specific primers designed based on annotated results from the transcriptome database. The PCR products were separated using the GeneJET™ Gel Extraction Kit (Thermo Scientific), and the purified DNA fragment was subcloned into the pCE2TA/Blunt-Zero vector (Vazyme Biotech, Nanjing, China) for sequencing. The primers used for cloning are listed in Supplementary Table 2.

### Expression of Limonene-3-Hydroxylase in *Saccharomyces cerevisiae* and Enzyme Assay

The limonene-3-hydroxyLase open read frame (ORF) was cloned into the vector pYeDP60 using the ClonExpress II One Step Cloning Kit (Vazyme Biotech, Nanjing, China). The obtained pYeDP60-L3OH was introduced into *Saccharomyces cerevisiae* WAT11 using a transformation kit (Frozen-EZ Yeast Transformation II Kit, ZYMO RESEARCH CORP.). The empty vector was used as a control. Transformation was plated on SGI medium containing 20 g/L glucose, 6.7 g/L yeast nitrogen base without amino acids, 1 g/L bacto casamino acids (Difco), 15 g/L agar, and 40 mg/L DL-tryptophan.

A single colony from SGI medium was grown in 50 mL of SGI liquid medium at 30°C for 12 h. The cells were centrifuged at 1630 × *g* for 5 min and resuspended in SGI medium containing 20 g/L galactose. The suspension was diluted OD600 of 0.4 and induced at 16°C for 12 h. Limonene solution was added to the culture to a final concertation of 0.2 mM. The reaction was stopped by sonication for 15 min for 12 h. The products from each reaction were extracted with 200 μL n-hexane. After the enzyme reactions, the glass vial was placed in a freezer (–80°C) for 2 h (Zhou et al., 2016) the upper organic phase was transformed into a new 2 mL glass vial containing a conical glass insert (while the still frozen aqueous phase of the original glass vial was discarded) and analyzed by GC-MS. Negative controls were generated by heating the reconstitution mix for 5 min at 100°C. The GC-MS column is described above. The injection temperature was 250°C and the solvent was delayed for 3 min. The oven was set at a temperature program of 50°C for 2 min, increased at 12°C/min to 100°C, and held for 25 min (Liu L. et al., 2021).

### Expression of Chavicol Methyl Transferase in *Escherichia coli* and Enzyme Assay

The chavicol methyl transferase ORF was cloned into pET28a to express CVOMT-His recombinant proteins. The constructs were transformed into *Escherichia coli* BL21 cells (TIANGEN, Beijing) using the heat shock treatment method at 42°C for 60 s. The transformation was plated on LB medium containing 100 ng/L kanamycin.

Single colonies were used to inoculate 5 mL of LB medium containing 100 ng/L kanamycin. One milliliter of the culture was transferred into 100 mL of the same medium and continued to grow at 37°C to reach an OD600 of approximately 1.0. Protein expression was induced by adding isopropyl β-thiogalactopyranoside to a final concertation of 1 mM. After 24 h incubation at 16°C, the cells were harvested by centrifugation (15 min at 4°C and 5,000 rpm). The cells were resuspended in 5 mL lysis buffer containing 10 mM/L Tris-HCl (pH 8.0), 200 mM NaCl, and 5% (v/v) glycerine. The suspension was transferred to an ultrasonic cell disruptor system and ultrasonically disrupted for 25 s each with 35 s on ice between disruptions. The mixture was centrifuged for 30 min at 4°C and 12,000 rpm and the supernatant was carefully removed (Liu C. et al., 2021).

Enzyme assays were performed using 30 μL protein extraction, 10 mM chavicol and 10 m S-adenosylmethionine in buffer (50 mM Tris-HCl, pH 7.0, containing 10% glycerol, 14 mM 2-mercaptoethanol, 1 mM EDTA, and 10 mM NaCl) to final volume of 400 μL in a 2 mL glass vial. The mixture was incubated at 31^°^C for 1 h and agitated by slowly rotating the glass vials (Gang et al., 2002). After the enzyme reactions, the glass vial was placed in a freezer (–80°C) for 2 h. The reaction was stopped by sonication for 15 min. The products from each reaction were extracted with 200 μL n-hexane. After the enzyme reactions, the glass vial was placed in a freezer (–80°C) for 2 h (Zhou et al., 2016) the upper organic phase was transformed into a new 2 mL glass vial containing a conical glass insert (while the still frozen aqueous phase of the original glass vial was discarded) and analyzed by GC-MS. Negative controls were generated by heating the reconstitution medium n-hexane for 5 min at 100°C. The GC-MS column is described above. The injection temperature was 250°C and the solvent was delayed for 3 min. The oven was set at a temperature program of 50°C for 3 min, then increased at 3°C/min to 90°C, held for 0 min, increased at 10°C/min to 220°C, and held for 5 min.

## Results

### Morphological Difference of Two Chemotypes of *Agastache rugosa*

**FIGURE 1:**
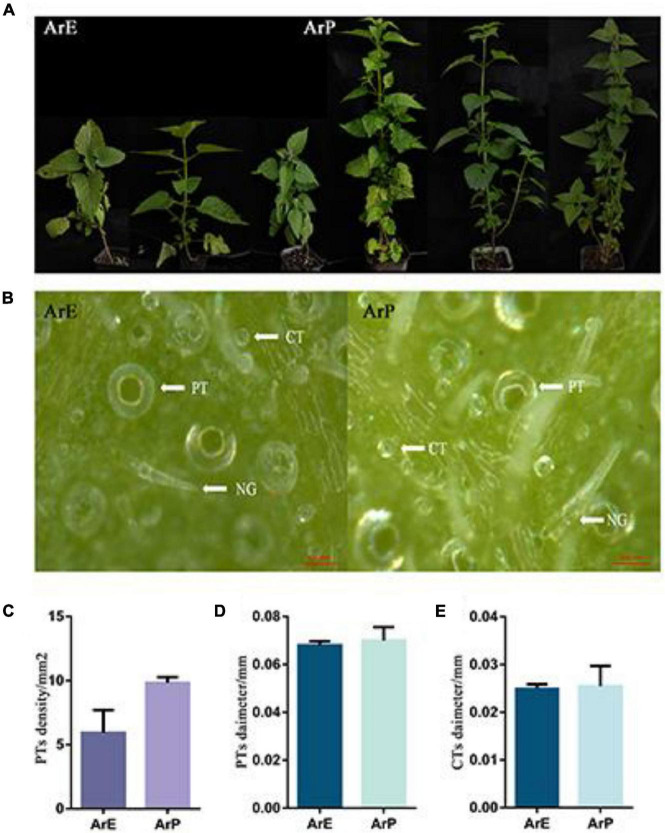
Morphological characterization of ArP and ArE of A. rugosa. **(A)** Photographs show three representative 12-week-old plants from the two chemotypes of *A. rugosa*. **(B)** Morphological features of trichomes on leaf surfaces, peltate trichome (PT), capitate trichome (CT), and non-glandular hairs (NG). **(C)** Peltate trichome density of ArE and ArP. Statistical significance was determined using the *t*-test (*P* < 0.05). **(D)** Peltate trichome diameter of ArE and ArP. Statistical significance was determined using the *t*-test (*P* > 0.05). **(E)** Capitate trichome diameter of ArE and ArP.

In addition to having a distinct chemical composition, the ArP and ArE of *A. rugosa* also have distinct morphological features. The morphological characteristics of ArP and ArE in *A. rugosa* are presented in Figure 1. The two chemotypes of *A. rugosa* are both perennial herbs in the Lamiaceae family, with four-prism stems and heart-ovate leaves with coarsely toothed edges. The plant height of the ArP was 1.79 times higher than that of the ArE, and the internode length was up to 1.36 times longer (Figure 1). The spike of the ArE grows at the top of the plant, while the spike of ArP grows at the top and axil of the plant, like that of peppermint and spearmint. The morphology of the flowers in the two chemotypes were similar: the corolla was pale blue-purple, and 2-lipped, the upper lip was straight, the apex was slightly emarginate, the lower lip was 3-lobed, and four stamens extended out of the corolla. The styles are subequal to stamens, filiform, and the apex equally 2-lobed (Supplementary Figure 1). The lower surface of the leaves of the two chemotypes of *A. rugosa* was evenly distributed with trichomes, and there were three types: peltate trichomes, capitate trichomes, and non-glandular hairs. The density of peltate trichomes in young leaves was higher than that in old leaves, and the diameter of peltate trichomes and capitate trichomes in young leaves was smaller than that in old leaves. There was a significant difference in the density of peltate trichomes between two chemotypes. The differences between ArP and ArE were slight in the diameter of peltate trichomes and capitate trichomes (Figure 1).

### Composition of Volatile Component in Different Organs of Two Chemotypes

As shown in Table 1, a total of 15 volatile compounds were detected in the two chemotypes by GC-MS analysis. The essential oil of ArP was partially dominated by (–)-pulegone, (–)-isomenthone, and (+)-limonene, which are monoterpenoids (Supplementary Figure 2). Thirteen compounds were detected in leaves. The total composition of (–)-pulegone, (+)-menthone, (–)-isomenthone, and (+)-limonene was up to 89% in leaves. Leaves had the highest levels of (–)-pulegone (59.01%), with significant quantities of (+)-limonene (9.04%) and only a small amount of (+)-menthone (<3%). There were fewer varieties of compounds in the stems than in the leaves. Compared to leaves, the composition of (+)-limonene in the stem was much higher than that in the leaves, which reached 22.27%. The composition of (+)-menthone in stems was also higher than that in the leaves. The content of (–)-pulegone was lower than that of the leaves. No essential oil was detected in the roots of ArP. Moreover, isopulegone was not detected in the essential oil of the stem. Estragole and methyl eugenol were not detected in ArP. In addition to monoterpenoids, caryophyllene, germacrene D, γ-elemene, β-cadinene, γ-muurolene, and α-cadinol were detected in ArP, which were not present in the essential oil of ArE.

**TABLE 1:** The content of the main component in volatile oil from different parts of *A. rugosa.*

ID	Compound	CAS	Foluma	Retention Time/min	Retention Index	Matching Index	Relative content (%)					
							ArP			ArE		
							Leaf	Stem	Root	Leaf	S tem	Root
1	β-Pinene	127-91-3	C_10_H_16_	10.675	979	854	0.67	0.61	–	–	–	–
2	(+)-Limonene	5989-27-5	C_10_H_16_	12.259	1030	921	9.04	22.27	–	2.68	5.83	–
3	(+)-Menthone	1196-31-2	C_10_H_18_O	17.769	1154	940	2.53	4.11	–	–	–	–
4	(–)-Isomenthone	18309-28-9	C_10_H_18_O	18.115	1164	911	21.44	35.99	–	–	–	–
5	(+)-Isopulegone	29606-79-9	C_10_H_16_O	18.483	1177	849	0.88	–	–	–	–	–
6	Estragole	140-67-0	C_10_H_12_O	19.140	1196	926	–	–	–	90.44	92.26	–
7	(–)-Pulegone	3391-90-0	C_10_H_16_O	20.137	1237	895	59.01	36.27	–	–	–	–
8	2-Cyclohexen-1-one, 3-methyl-6-(1-methylethylidene)-	491-09-8	C_10_H_14_O	22.160	1340	838	0.56	–	–	–	–	–
9	Methyleugenol	93-15-2	C_11_H_14_O_2_	23.143	1402	841	–	–	–	6.89	1.92	–
10	Caryophyllene	87-44-5	C_15_H_24_	23.463	1419	856	0.58	0.20	–	–	–	–
11	Germacrene D	23986-74-5	C_15_H_24_	24.360	1481	897	1.29	–	–	–	–	–
12	γ-Elemene	29873-99-2	C_15_H_24_	24.574	1433	873	2.11	0.56	–	–	–	–
13	β-Cadinene	523-47-7	C_15_H_24_	24.901	1518	807	0.40	–	–	–	–	–
14	γ-Muurolene	30021-74-0	C_15_H_24_	25.624	1477	814	1.06	–	–	–	–	–
15	α-Cadinol	481-34-5	C_15_H_26_O	26.572	1653	832	0.42	–	–	–	–	–

Only three compounds were detected in ArE; the main contents of the essential oil were estragole and caryophyllene. The composition of estragole was the highest in the stem and leaves of ArE, which was up to 90%. In addition, (+)-limonene was detected in the leaves and stems, while other monoterpenoids were not detected in ArE.

### RNA Sequencing and *de novo* Transcriptomic Assembly

**FIGURE 2:**
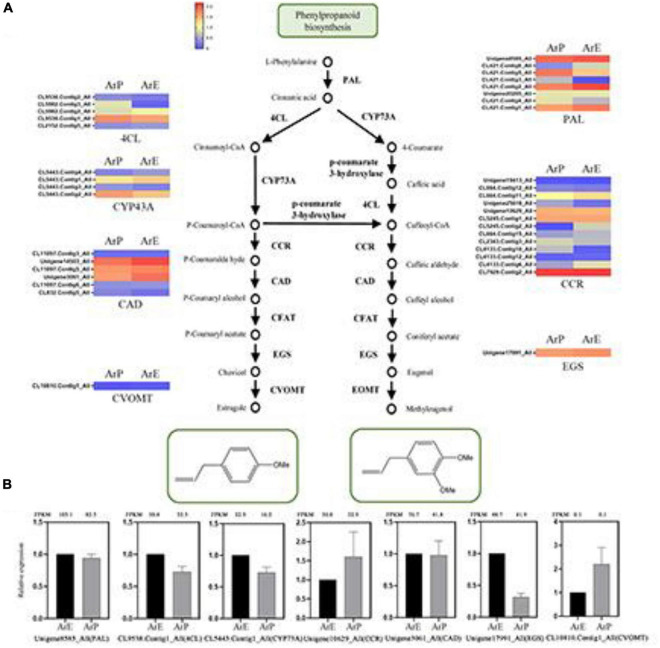
Schematic overview of the phenylpropanoids biosynthesis pathway and the expression of representative genes by RT-qPCR. **(A)** Schematic overview of the phenylpropanoids pathway in *A. rugosa* and heatmap of the corresponding enzymes involved in the pathway. The main enzymes are shown with the abbreviation throughout respective enzymatic steps. Enzyme abbreviations are PAL, phenylalanine ammonia-lyase; 4CL, 4-coumarate: CoA ligase; CCR, coumaroyl-coA reductase; CAD, coumaryl-aldehyde dehydrogenase; CFAT, caffeic aldehyde acyltransferase; EGS, eugenol synthase; CVOMT, chavicol methyl transferase; EOMT, eugenol methyl transferase. **(B)** The expression level of representative genes involved in estragole biosynthesis in *A. rugosa*.

We constructed separate cDNA libraries from the two chemotypes of the *A. rugosa* samples, and the cDNA was sequenced using the DNBSEQ platform (Mahmood et al., 2020). Six sequencing libraries were constructed, including three replicates for both estragole and pulegone chemotypes. Each sample produced at least 42 Mb clean reads and 6 GB of data. After filtering and removing the low quality, linker contamination, and high N content of unknown reads from the raw data, the percentage of clean reads was more than 91% and Q20 percentage was at least 95.3% for the six libraries (Supplementary Table 3). High-quality reads were selected for further analysis, and clean reads were assembled using the Trinity program. We then used Tgicl to cluster transcripts to remove redundancy to obtain unigenes, named “All-Unigene” (Haas et al., 2013). Finally, 100852 unigenes were assembled with a GC percentage of 41.37%, and the average length and N50 lengths were 1530 and 2323 bp, respectively (Supplementary Table 4). The length distribution of the unigenes is indicated in Figure 2, and 85.17% of all unigenes had lengths longer than 300 bp. Benchmarking Universal Single-Copy Orthologs (BUSCO) was used to evaluate the quality of the assembled transcripts (Simão et al., 2015). By comparing them with the conserved genes, it shows the integrity of the transcriptome assembly to a certain extent. The assembled evaluation results are shown in Supplementary Figure 3.

### Functional Annotation of Unigenes

The gene functions of the assembled transcripts were annotated using public databases (Supplementary Figure 4). The results showed that 39499 unigenes were annotated in five public databases. GO, KOG, and KEGG assignments were used to classify the functions of the assembled unigenes of *A. rugosa* (Supplementary Table 5). For GO annotation, all unigenes were divided into 25 groups. Among them, general function prediction only (11691), signal transduction mechanisms (7663), and posttranslational modification, protein turnover, and chaperones (4697) were the largest groups. For KEGG pathway analysis, 32084 unigenes were mapped to metabolic pathways, among which “metabolism” was the largest category.

### Analysis of Differential Gene Expression

Using the DEseq2 method with *Q*-value (Adjusted *P*-value) ≤ 0.05, 4590 differentially expressed genes (DEGs) were detected in the two chemotypes of *A. rugosa*. In the ArE and ArP comparison groups, 2,635 and 1,955 genes were upregulated and downregulated, respectively (Supplementary Figure 5). To further explore the possible role of DEGs, we conducted GO and KEGG enrichment experiments. The enrichment degree of the GO Term is displayed in the form of a histogram. By default, the top 20 GO Term with the smallest *Q*-value or the selected GO Term (sorted by *Q*-value, up to 60) are plotted. “ADP binding,” “auxin-activated signaling pathway,” and “beta-glucosidase activity” were the most significantly enriched terms in “molecular function.” The bubble chart of KEGG pathway enrichment results showed that 20 pathways were significantly enriched in “E vs. P.” Among them, “Plant-pathogen interaction” and “Plant hormone signal transduction” enriched the largest number of differentially expressed genes (Supplementary Figure 5). Using the heatmap function in R software to perform hierarchical cluster analysis on the FPKM values of differentially expressed genes, the three replicates of different chemotypes of samples are clustered into one type.

### Identification of Transcription Factors

Plant transcription factor prediction uses the Plant TFDB database and compares it with Pfam23.0, using the hmmpfam of the HMMER program, and searches for protein domains to identify genes encoding transcription factors. A total of 2918 unigenes were annotated as transcription factors of different families. Among them, AP2/ARF, WRKY, bHLH, and bZip family transcription factors are believed to be related to the synthesis of terpenoids, and AP2/ARF and MYB family transcription factors are considered to be related to the growth and development of glandular trichomes.

### Candidate Genes Involved in Monoterpenoids and Phenylpropanoids Biosynthesis

**FIGURE 3:**
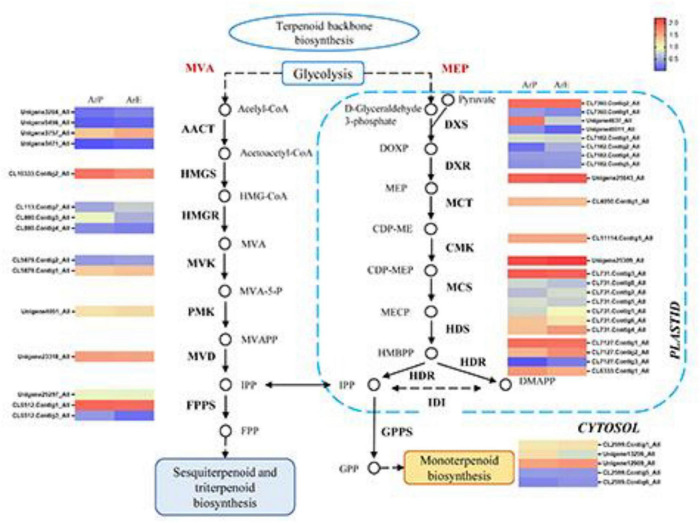
Schematic overview of the MVA and MEP pathway in *A. rugosa* and heatmap of the corresponding enzymes involved in the pathway. The main enzymes are shown with the abbreviation throughout respective enzymatic steps. Enzyme abbreviations are AACT: acelyl-CoA C-acetyltransferase; HMGC, hydroxymethylglutaryl-CoA synthase; HMGR, hydroxymethylglutaryl-CoA reductase; MVK, mevalonate kinase; PMK, phosphomevalonate kinase; MVD, diphosphomevalonate decarboxylase; FPPS, farnesyl pyrophosphate synthase; DXS, 1-deoxy-D-xylulose-5-phosphate synthase; DXR, 1-deoxy-D-xylulose-5-phosphate reductase; MCT, 2-C-methyl-D-erythritol 4-phosphate cytidylyltransferase; CMK, 4-diphosphocytidyl-2-C-methyl-D-erythritol kinase; MCS, 2-C-methyl-D-erythritol 2,4-cyclodiphosphate synthase; HDS, (E)-4-hydroxy-3-methylbut-2-enyl-diphosphate synthase; HDR, 4-hydroxy-3-methylbut-2-en-1-yl diphosphate reductase; IDI, isopentenyl-diphosphate Delta-isomerase; GPPS, geranyl diphosphate synthase.

To explore the regulatory mechanisms for the accumulation patterns of different terpenoids in *A. rugosa*, the expression profiles of genes involved in terpenoid biosynthesis were analyzed. In the ArE and ArP comparison groups, 46 DEGs for the biosynthesis of terpenes were identified. Most of the genes exhibited high transcriptome expression levels, encoding key enzymes in the MEP and MVA pathways (KEGG entry ko00900). The expression levels of different unigenes between the two chemotypes were different, but the unigene expression level of the MEP pathway was higher than that of the MVA pathway (Figure 3).

**FIGURE 4:**
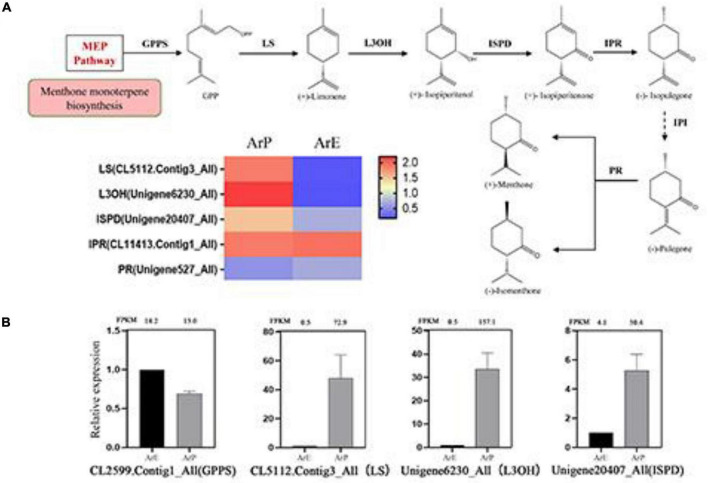
Schematic overview of the monoterpenoid biosynthesis pathway and the expression of representative genes by RT-qPCR. **(A)** Schematic overview of the monoterpenoid pathway in *A. rugosa* and heatmap of the corresponding enzymes involved in the pathway. The main enzymes are shown with the abbreviation throughout respective enzymatic steps. Enzyme abbreviations are GPPS, geranyl diphosphate synthase; LS, limonene synthase; L3OH, limonene-3-hydroxylase; ISPD, isopiperitenol dehydrogenase; IPR, isopiperitenone reductase; IPI, isopiperitenone isomerase; PR, pulegone reductase. **(B)** The expression level of representative genes involved in menthol biosynthesis in *A. rugosa*.

Pulegone is the main constituent of monoterpenoids in the ArP of *A. rugosa*, which is synthesized by a series of enzymatic reactions in the peltate trichomes on the surfaces of the plant. The biosynthetic pathway and enzyme catalysis mechanism of pulegone and menthone have been extensively studied in *Schizonepeta* (Liu et al., 2018). Using actin as the reference gene, four genes and their orthologous genes catalyze the biosynthesis of (–)-pulegone from (+)-limonene were identified in *A. rugosa* for the first time (Figure 4). Only one unigene encoding one enzyme was selected and identified based on sequence similarity, with the unigene: CL5112.Contig3_All encoding limonene synthase (LS), Unigene6230_All encoding limonene-3-hydroxylase (L3OH), Unigene20407_All encoding isopiperitenone dehydrogenase (ISPD), and Unigene527_All encoding pulegone reductase (PR). The expression levels of enzymes involved in monoterpenoid biosynthesis of ArP were higher than those of ArE, except for PR (Figure 4).

Estragole is the representative compound of ArE. The biosynthesis of estragole has also been reported (Yu and Jez, 2008). Based on sequence similarity, there were seven genes identified which catalyze the biosynthesis of phenylpropanoid from L-phenylalanine, with the unigene: Unigene8585_All encoding phenylalanine ammonia-lyase (PAL), CL9538.Contig1_All encoding 4-coumarate:CoA ligase (4CL), CL5443.Contig1_All encoding CYP73A, Unigene10629_All encoding coumaroyl-CoA reductase (CCR), Unigene3061_All encoding coumaryl-aldehyde dehydrogenase (CAD), Unigene17991_All encoding eugenol synthase (EGS), and CL10810.Contig1_All encoding chavicol methyl transferase (CVOMT). The expression levels of genes involved in estragole biosynthesis of ArE were higher than those of ArP, except for CCR and CVOMT (Figure 2).

### Quantitative Real-Time PCR Validation of Differentially Expressed Genes

To validate the expression pattern of monoterpenes and phenylpropanoid biosynthesis, qRT-PCR was conducted to examine the expression levels of 14 unigenes in the two chemotypes. The expression levels of these selected genes from qRT-PCR analyses were generally consistent with the deducted from their fragments per kilobase per million mapped data from the RNA-seq. These results confirmed the reliability of the transcriptomic profiling data.

### Expression and Enzyme Assays of Limonene-3-Hydroxylase and Chavicol Methyl Transferase

**FIGURE 5:**
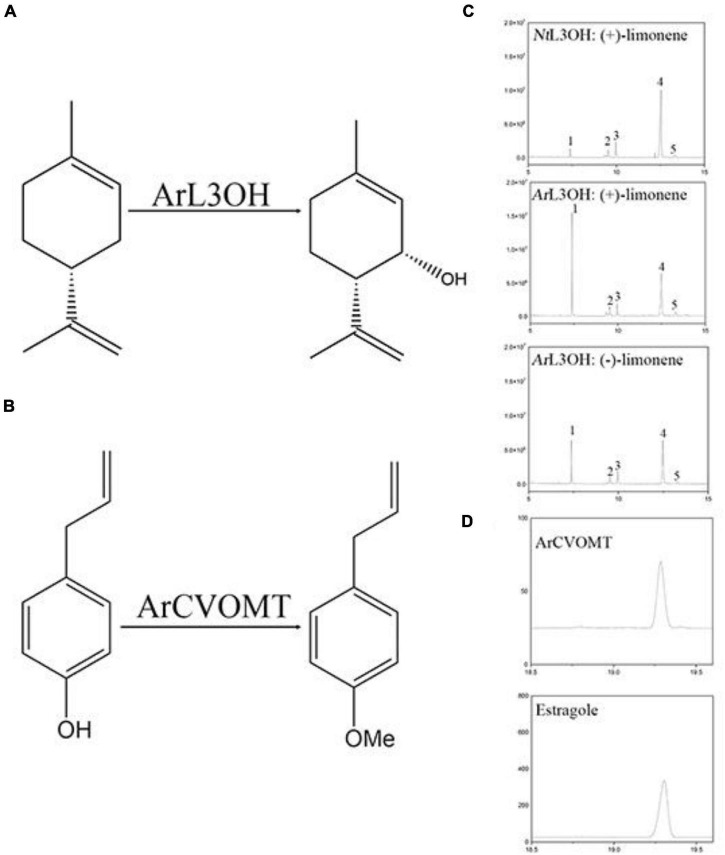
Functional characterization of L3OH and CVOMT. **(A)** Reaction of limonene to isopiperitenol catalyzed by limonene-3-hydroxylase from *A. rugosa*. **(B)** The reaction of chavicol to estragole catalyzed by chavicol menthyl transferase. **(C)** GC chromatograms of products from *Nt*L3OH and *Ar*L3OH with limonene. 1, the peak of limonene; 2, the peak of C1-OH product; 3 and 4, unknown; 5, the peak of tenol. **(D)** A GC chromatogram of products from CVOMT with chavicol; b, GC chromatograms of the standard of estragole.

The sequencing information extracted from the transcriptome data of L3OH was used to amplify the corresponding full-length cDNA (NCBI accession number: OK649356). To examine the function of the candidate gene in menthol biosynthesis in *A. rugosa*, we expressed AgL3OH in *S. cerevisiae*. We conducted enzyme assays *in vitro*, in which the reaction was fed with (–)-limonene and (+)-limonene. The reaction was detected and analyzed using GC and chiral-GC. Since there was no authentic standard available, it can be speculated to be a product of limonene according to our previous report (Liu L. et al., 2021). The results showed that the *Ar*L3OH enzyme converted (+)-limonene and (–)-limonene into (–)-isopiperitenol and (+)-isopiperitenol as the major products (Figure 5).

The full-length cDNA (NCBI accession number OK649357) of *CVOMT* was amplified by PCR. The recombinant *CVOMT* protein was transferred into the expression vector pET28a. We expressed *CVOMT* in *E. coli*. We performed enzyme assays *in vitro*. Chavicol was added to the reaction and detected using GC. Compared with the retention time of the standard, the results showed that the *CVOMT* enzyme converted chavicol into estragole under the action of S-adenosylmethionine (Figure 5).

## Discussion

There are different secondary metabolites among different chemotypes in Lamiaceae, Asteraceae, and Lauraceae, such as *Perilla frutescens* and *Artemisia annua* (Czechowski et al., 2018; Zhou et al., 2021), which generate multiple pharmacological activities, such as *Cinnamomu porrectum* and *Eugenia uniflora* (Figueiredo et al., 2019; Qiu et al., 2019). Therefore, chemotype formation is the focus and complexity in research on plants, especially in medicinal plants.

At present, *A. rugosa* is classified as a pulegone chemotype, estragole chemotype, and methyl eugenol chemotype (ArM) based on their different oil constituents (Li et al., 2013), which may have different pharmacological effects. Pulegone of *A. rugosa* has antimicrobial activity, whereas the estragole of *A. rugosa* has strong nematocidal activity (Li et al., 2013; Gong et al., 2016). Therefore, studying the formation and mechanism of chemotypes plays an important role in the utilization of *A. rugosa*. However, only LS and PR in the pulegone biosynthesis pathway has been characterized in *A. rugosa* (Maruyama et al., 2002; Liu C. et al., 2021). In this study, we compared the morphological differences, metabolite constituents, and transcriptomic data between the two chemotypes of *A. rugosa*.

The chemotypes of one species are a general phenomenon in plants, leading to morphological differences (Czechowski et al., 2018). Plant height and internode length were the main morphological differences between the high -and low-artemisisnin chemotypes in *Artemisia annua* (Czechowski et al., 2018). Different stem types exist in different chemotypes of *Thymus quinquecostatus* (Kim et al., 2020). The morphological differences of various chemotypes in mints mainly focused on inflorescence characteristics. Our study found that plant height and internode length were the main differences between the two chemotypes in *A. rugosa*.

For most plants, the main compounds of different chemotypes in the same species present similar skeletal structures. Terpenoids are the main compounds of different chemotypes in *Origanum vulgare* (Wêglarz et al., 2020). There are also a few plants with different chemotypes of the principal components that have different skeletal structures, such as *Ocimum* (Anand et al., 2019). Compared with the metabolites of ArP and ArE by GC-MS, monoterpenoid is the main constituent in the essential oil of ArP, while phenylpropanoids are the main constituents of the essential oil of ArE, except for limonene, which is the only common component in the essential oil of these two chemotypes. We also detected methyl eugenol in ArE, which is a possible transition chemotype between ArP and ArM. However, there was no downstream product after limonene in ArE. This is consistent with the expression levels of genes involved in menthone biosynthesis of ArE. *LS*, *L3OH*, and *IPD* were highly expressed in ArP, *and GPPS* was highly expressed in both chemotypes of *A. rugosa*. The unigenes related to estragole biosynthesis in ArP were downregulated, except for *CCR* and *CVOMT.* We speculate *that CCR* and *CVOMT* are not only involved in estragole biosynthesis, but also participate in physiological biochemical processes (Wengenmayer et al., 1976; Gang et al., 2002). As expected, and we found that L3OH could catalyze both (+)-limonene and (–)-limonene to (+)-isopiperitenol and (–)-isopiperitenol. Apart from the main product, the byproducts were discovered as described (Liu L. et al., 2021). The functional characterization of CVOMT was identified. Under the catalytic action of CVOMT and S-adenosylmethionine, chavicol was transferred to estragole. Therefore, in addition to improving our understanding of different chemotypes in the Lamiaceae, our results highlight targeted genes for volatile oil biosynthesis in *A. rugosa*, which could benefit the genetic improvement of *A. rugosa* and other Lamiaceae species. However, one of the key points in chemotype formation is the regulation of specialized gene expression in specialized chemotypes, which requires further research.

## Data Availability Statement

The datasets presented in this study can be found in online repositories. The names of the repository/repositories and accession number(s) can be found below: https://www.ncbi.nlm.nih.gov/, PRJNA774149; https://www.ncbi.nlm.nih.gov/, OK649356; and https://www.ncbi.nlm.nih.gov/, OK649357.

## Author Contributions

JD performed functional characterization, content integration, and manuscript composition. GL performed data analysis, manuscript composition, qPCR experiments, and manuscript composition. CL assisted in the qPCR experiments. PZ and SD performed the morphological experiments. MS and ZJ conducted oil collection. YS performed GC-MS detection and analysis. QW and CL were responsible for the overall concept, design of the experiments, data integration, and manuscript revision. All authors contributed to the article and approved the submitted version.

## Conflict of Interest

The authors declare that the research was conducted in the absence of any commercial or financial relationships that could be construed as a potential conflict of interest.

## Publisher’s Note

All claims expressed in this article are solely those of the authors and do not necessarily represent those of their affiliated organizations, or those of the publisher, the editors and the reviewers. Any product that may be evaluated in this article, or claim that may be made by its manufacturer, is not guaranteed or endorsed by the publisher.
