# Supplementary material for: Comparison of Pulegone and Estragole Chemotypes Provides New Insight Into Volatile Oil Biosynthesis of Agastache rugosa
Source: Front Plant Sci. 2022 Apr 6;13:850130. doi: 10.3389/fpls.2022.850130 (PMC9019551; doi:10.3389/fpls.2022.850130)
Supplement: Supplementary file 1 [file Data_Sheet_1.docx]

# Supplementary Figures


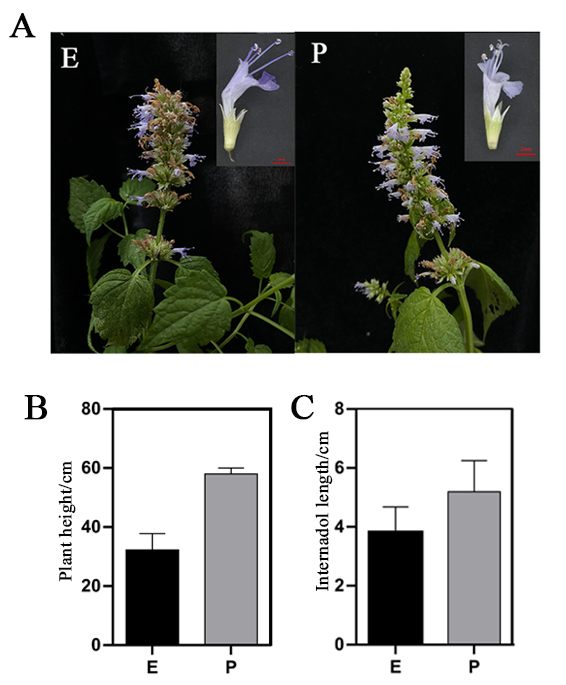


**Supplementary Figure 1**. (A), Morphology of spikes, stamen, and pistil of each two chemotypes of *A. rugosa*. (B), Plant height of ArE and ArP. (C), Internode length of ArE and ArP.


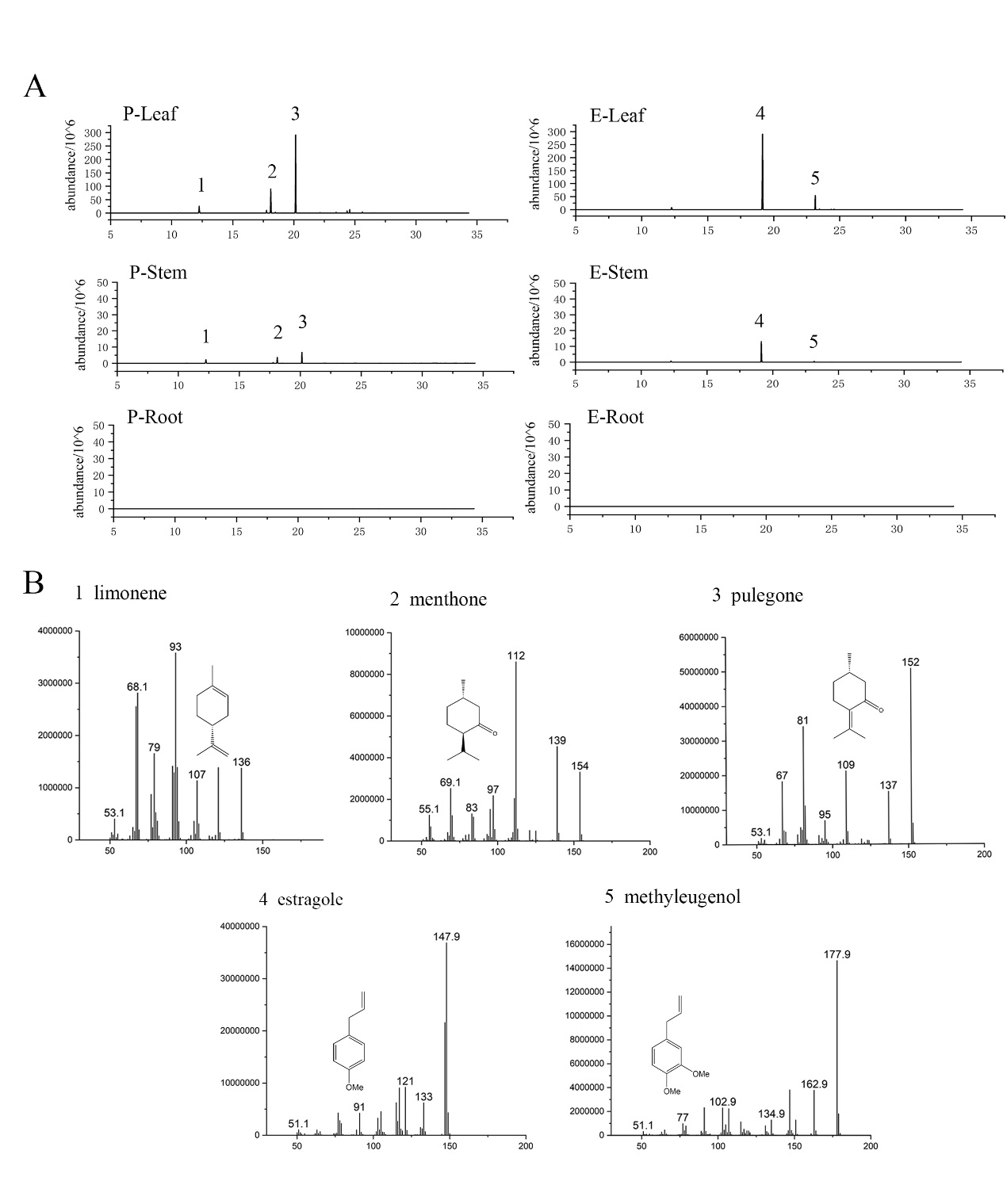


**Supplementary Figure 2.** The difference of components in different chemical types of plants and main compounds detected by GC-MS.(A), Chromatographic ion peak map of different tissue (leaf, stem, root, PTs of leaves) from each two chemotypes of A. rugosa(B), Secondary mass spectrogram and chemical formula of 5 compounds in A. rugosa.


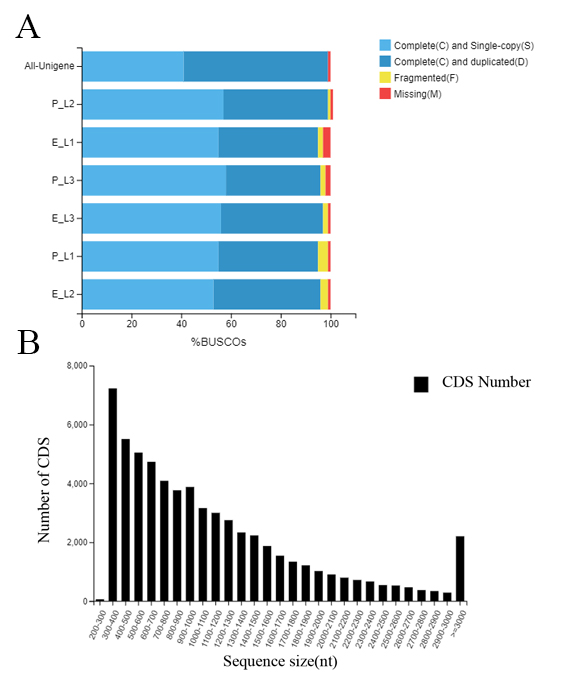


**Supplementary Figure 3.** (A), Length distribution of assembled CDS. (B), Evaluation results of BUSCO based on evolutionarily-informed expectations of gene content of near-universal single-copy orthologs.


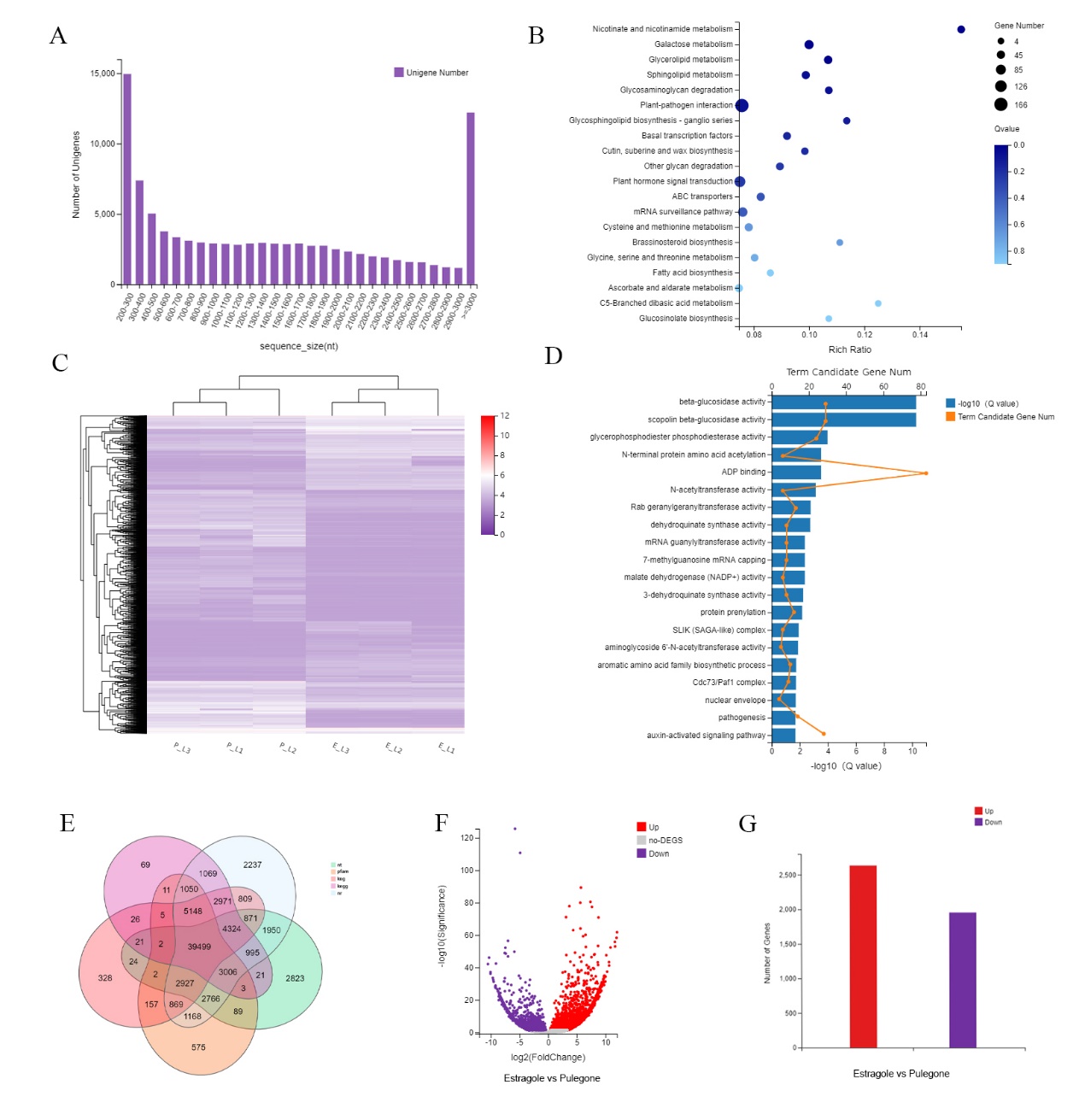


**Supplementary Figure 4.** Annotation of assembled unigenes. (A), KEGG annotation of assembled unigenes. (B), KOG annotation of assembled unigenes. (C), GO annotation of assembled unigenes. (D), TF annotation of assembled unigenes.


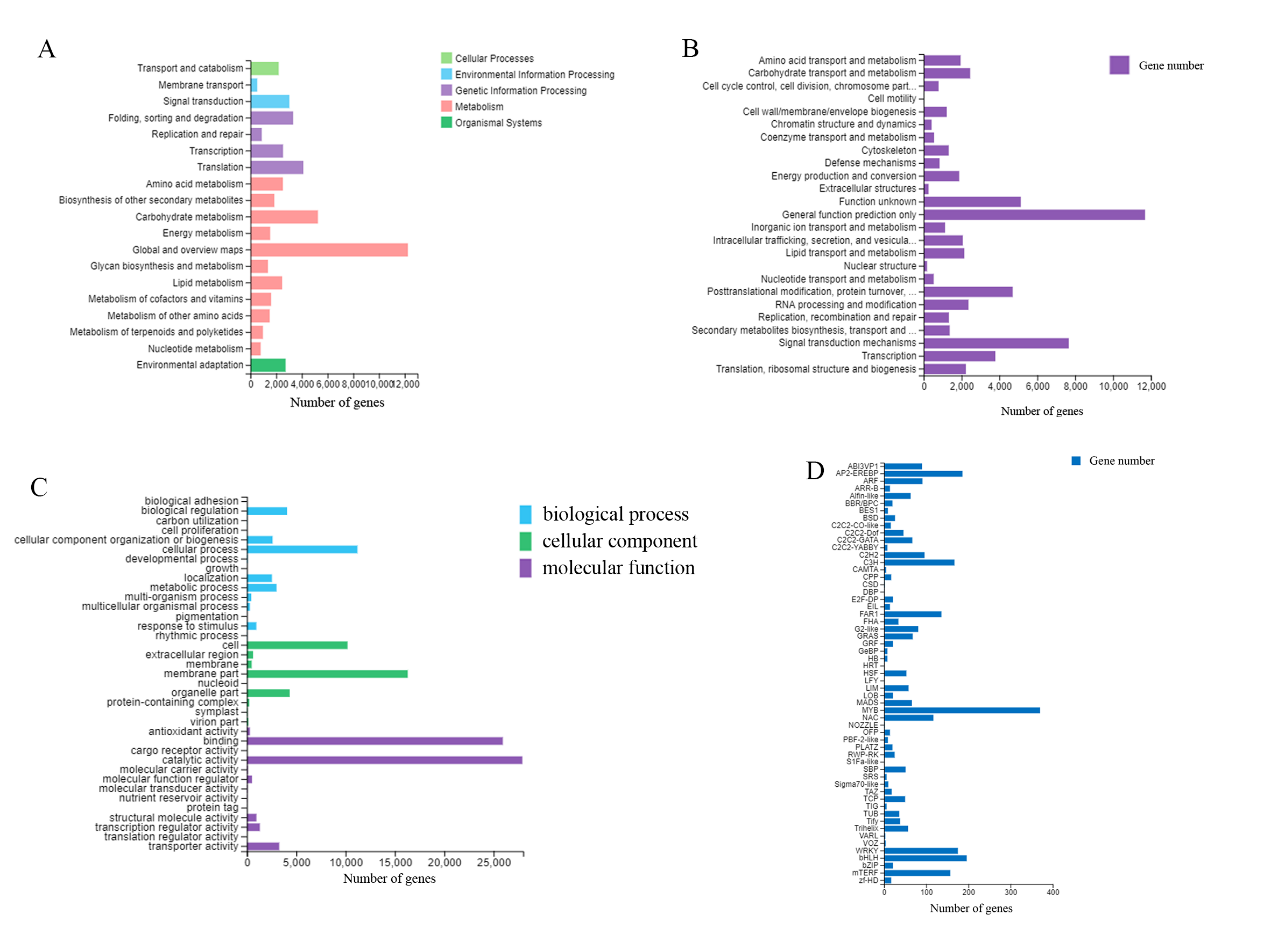


**Supplementary Figure 5.** **RNA sequence and de novo transcriptomic**. (A), Length distribution of unigenes. (B), KEGG bubble diagram of DEGs.(C),Heatmap of DEGs cluster analysis.(D), GO bar chart of DEGs.(E), Annotation information of the Venn diagram. (F), Volcano plot of DEGs. (G), DEG numbers in the comparisons of pulegone chemotype and estragole chemotype. DEG: Differentially Expressed Gene.

# Supplementary Tables

**Supplemetary Table 1.** Primer information for qRT-PCR.

| Gene | Sequence (5’-3’) |
| --- | --- |
| GPPS | F: AGCATTCCTCTCACTTTCGC  R: TCTTTTTCCTTCAACTCCCA |
| LS | F: CGACTCTCTGTACGAATACCAC  R: TCATCTTCTTCCACATCTCTCT |
| L3OH | F: GGCAGCGTCATCAAGGATTA  R: CCAGTTTGGAGGAAGGAAAG |
| ISPD | F: GAGGAGCAGGTAAAGTCCATGATAGAA  R: ATCACGTAGTCCGTCAAGTTGGAAGCG |
| PAL | F: AAATCTCTCAGGCGGGAGGAAC  R: TGGCATAAGGCGACCAAGTAGG |
| 4CL | F: TCCAACCACCTCCCTCTCCA  R: CGTCGCCTTTCCTAATTCCG |
| CYP73A | F: TGGGGCATTGCTGAGCTAGT  R: GTCGTGGAGGTTCATGTGGG |
| CCR | F: ATGAGCGAGAAAGATGGA  R: CCTGAAGGTTTTGGACGG |
| CAD | F: TGAGGTGGGAAAGAAGGT  R: GCACACAGAAGTGGAGCA |
| EGS | F: CTTCCAAGACAAACCTCCTC  R: TCAACTTCCTTCATTACCGA |
| CVOMT | F: TCACCACCGAGCACCACCTC  R: CTTTCCAAGCCAGCCACAAC |

**Supplemetary Table 2.** Primers used for the construction of recombaint plasmids.

| **Gene** | **Sequence (5’-3’)** |
| --- | --- |
| **L3OH** | **F:** **ACACACTAAATTACCGGATCCATGGAGGTCGTGGAGGTTT**  **R:** **TGGGAGATCCCCCGCGAATTCTTAAGGATTGTAGGGC** |
| **CVOMT** | **F: GTCGTTTACCCAGCGCCTAGGATGGCGTTGCAAAATGGAGA**  **R: CGTTCGAACAGCTGCCTCGAGTTATTGTGTGTATCTCGTTAGTGGTGTT** |

**Supplemetary Table 3.** Summary of DNBSEQ sequencing data.

| Sample^a^ | Total Raw Reads(M) | Total Clean Reads(M) | Total Clean Bases(Gb) | Clean Reads Q20(%)^b^ | Clean Reads Q30(%) | Clean Reads Ratio(%) |
| --- | --- | --- | --- | --- | --- | --- |
| E_L1 | 47.33 | 43.43 | 6.52 | 95.97 | 90.58 | 91.78 |
| E_L2 | 47.33 | 43.38 | 6.51 | 95.75 | 90.12 | 91.66 |
| E_L3 | 45.99 | 42.08 | 6.31 | 95.30 | 89.25 | 91.51 |
| P_L1 | 47.33 | 43.55 | 6.53 | 95.72 | 90.07 | 92.02 |
| P_L2 | 47.33 | 43.48 | 6.52 | 95.96 | 90.55 | 91.86 |
| P_L3 | 47.33 | 43.51 | 6.53 | 95.72 | 90.09 | 91.93 |

^a^ E and P represent libraries constructed by estragole and pulegone chemotypes of samples.L indicates that the leaf is the sampling tissue.Numbers indicate three biological replicates;b Q20 percentage represents percentage of bases with a Phred value >20.

**Supplemetary Table 4.** Statistics of assembly data.

| All-unigene number | 100852 |
| --- | --- |
| Total length(bp) | 154,320,997 |
| Mean length(bp) | 1530 |
| N50(bp) | 2323 |
| GC(%) | 41.37 |

**Supplemetary Table 5.** Functional annotations of *A.rugosa* unigenes.

| Annotation Database | Number of Unigenes | Percentage (%) |
| --- | --- | --- |
| Nr | 71659 | 71.05% |
| Nt | 59323 | 58.82% |
| Swissprot | 55069 | 54.60% |
| KEGG | 58220 | 57.73% |
| KOG | 57983 | 57.49% |
| Pfam | 57277 | 56.79% |
| GO | 53449 | 53.00% |
| Total unigenes | 75876 | 75.23% |

Nr: National Center for Biotechnology Information (NCBI) non-redundant protein database; KOG: eukaryotic Orthologous Group; KEGG: Kyoto Encyclopedia of Genes and Genomes; GO: Gene Ontology.
